# Valuating marine knowledge: Heterogeneous collaborations at the Concarneau marine station

**DOI:** 10.1007/s40656-025-00683-5

**Published:** 2025-07-24

**Authors:** Tanja Bogusz

**Affiliations:** Department of Socioeconomics, Hamburg University, Hamburg, Germany

**Keywords:** Democratic experimentalism, Marine social sciences, Material politics, Pragmatism, Social cohesion, STS

## Abstract

Marine stations have long been explored by science and technology studies (STS) and the humanities as boundary objects between the field and the lab. However, through their position, they embrace rather three domains which have been separated by the modern organization of knowledge–*sea, science*, and *society*–not only epistemically, but also physically. In contrast to time-limited marine expeditions or pure laboratory work, marine stations enact “science with their feet in the water” while situated within concrete local societies. Therefore, many marine stations provide multiple ways of valuating the relation between the sea and society. However, in an era of considerable polarization regarding a sustainable future for coastal communities, valuating marine knowledge is a socially complex endeavor. Based on a five-month ethnography of the world’s oldest existing institution of this kind, the Station Marine de Concarneau in Brittany, France, this paper discusses its practical enactment of heterogeneous values associated with marine knowledge. The paper, *first*, introduces the Concarneau station, its particular research profile, and its local exposure. *Second*, an experimentalist approach based on pragmatism and STS is explored in order to rethink current research on the valuation of socio-marine cohesion. *Third*, two types of valuating marine knowledge through heterogeneous collaborations at the station are explored: a) socio-technical and b) socio-epistemic. *Finally*, the paper deduces the importance of marine stations for inter- and transdisciplinary collaboration within the global transformation of sea-society relations.

## Introduction

**Fig. 1:**
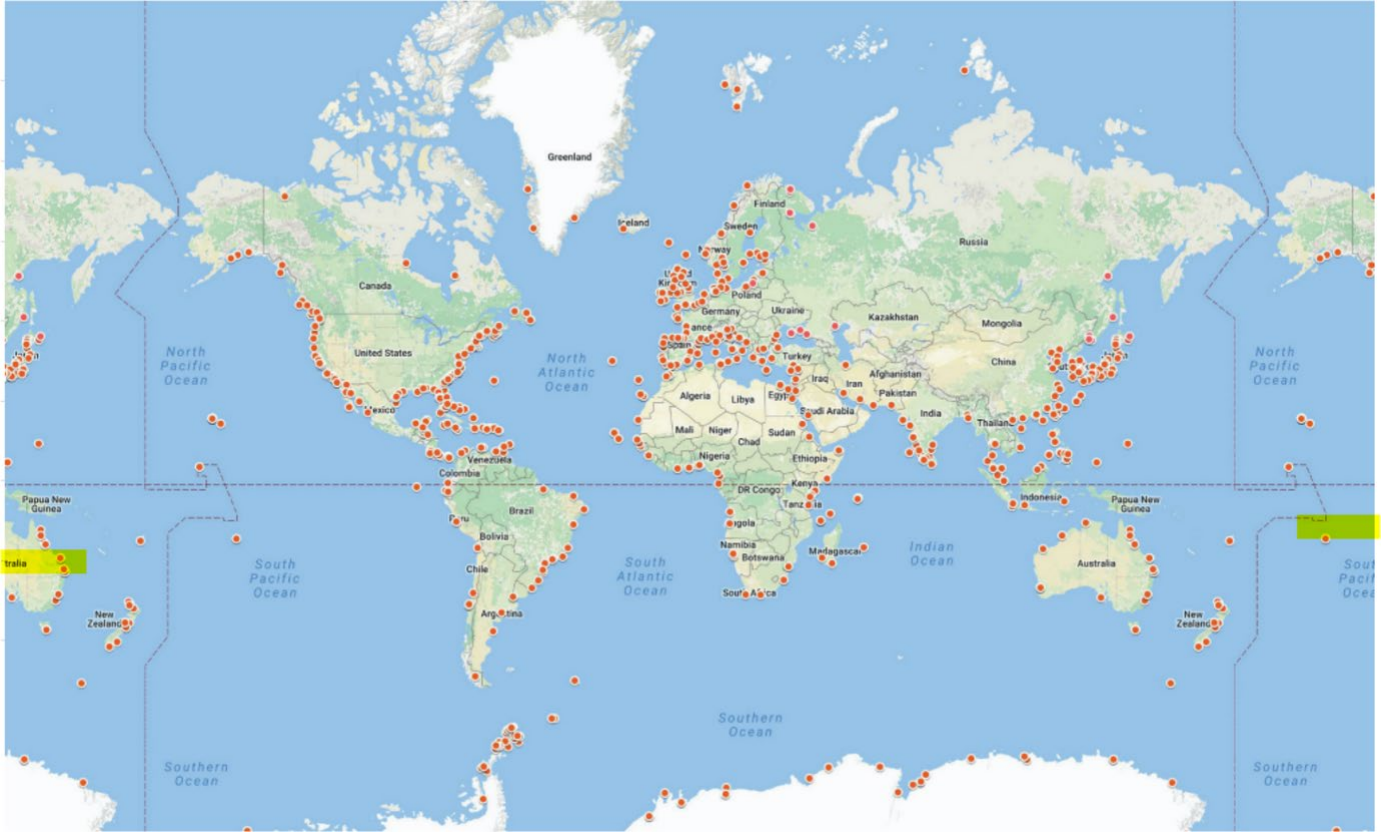
World distribution of marine stations Source: Global Ocean Science Report 2017: 65

Marine stations tackling the ongoing crisis of the oceans are excellent sites for ethnographic inquiry in the Anthropocene. Through their position, they embrace three domains which have been separated by the modern organization of knowledge–*sea, science,* and *society*–not only ontologically, but also physically. However, while historical science studies and the humanities have examined marine stations as boundary objects between field and lab, i.e., as places that interconnect sea and science (Kohler, 2002; Philp, 2023), they have overlooked these stations’ interconnectedness with society. Surprisingly, contemporary marine stations have also been understudied in this regard, despite their omnipresence (Fig. 1).

Despite their impressive global distribution, there is still a lack of systematic and comparative studies on marine-station activities in relation to their coastal communities. While there is a rich and fascinating body of literature on the history of specific stations, exploring the itineraries of their leading creators and their research achievements, strategies, and networks (Kohler, 2002; Groeben, 2004; De Bont, 2009; Dayrat, 2016; Lamy et al., 2016; Steiner, 2020), it is harder to find contemporary research on this area (Geissler & Kelly, 2016; Heggie, 2016). Yet at the science level, marine stations are key players in facilitating access to and the long-term observation of the ocean, as well as onshore experiments and training. Accordingly, as the 2017 Global Ocean Report suggests, they are “defined as field stations where scientific research and observation of marine organisms, ecosystems, and environments are carried out” (IOC UNESCO, 2017 p. 2). Indeed, as a recent film by the French National Centre for Scientific Research (CNRS) tells us, marine stations enact “science with their feet in the water.”^1^ Like other biological field stations situated in coastal areas, they valuate “landscapes and labscapes” (Kohler, 2002). The ocean, through its physical extension on the globe, represents the most foundational landscape^2^ of planetary life, one which is, in the current era of the Anthropocene, under fundamental threat. The same applies to the communities living in and near it.

In view of the current anthropogenic pressure on the oceans, causing their ecological degradation, sea-level rise, and flooding, a more comprehensive perspective on marine stations, including their engagement with their surrounding marine collectives, seems timely. Inspired by pragmatist theories of knowledge and of science and technology studies (STS), this paper explores the marine station as a facility enabling collaboration across heterogeneous marine collectives. Here, the valuation of marine knowledge is practiced in a situated environment where actors follow multiple, at times disparate, ends. Such valuation often happens through modes of cooperation that are not driven by shared goals. Rather, they are instilled by a pragmatist tenet, according to which cooperation requires the art of “dealing with people *unlike ourselves*” (Sennett, 2012, p. ix, my italics). This art is practiced on an everyday and experimental basis, often channelled through material entities, rather unnoticed, but nonetheless crucial in times of eco-social polarization.

Cooperation across differences, though constantly acclaimed in the political symphony of sustainable science, is socially complex. Through an in-depth qualitative examination of a specific station, I will discuss the reciprocal interwovenness of sea, science, and society as ontological entities. Marine stations, being situated within coastal communities and with their feet in the water, are excellent cases for rethinking practices of valuation through the lenses of marine knowledge. Based on a five-month ethnography of the world’s oldest existing institution of this kind, the Station Marine de Concarneau in Brittany, France, the paper discusses marine knowledge and experience associated with the ocean. The paper, *first*, introduces the Concarneau station, its research profile and local situation. *Second*, an experimentalist approach based on pragmatism and STS is explored in order to rethink current research on the valuation of socio-marine cohesion. *Third*, two types of valuating marine knowledge through heterogeneous collaborations are explored: a) socio-technical and b) socio-epistemic. *Finally*, the paper concludes on the mutualizing potential of these valuations and of social studies of marine stations within the global transformation of sea-society relations.

## The Concarneau Marine Station

**Fig. 2:**
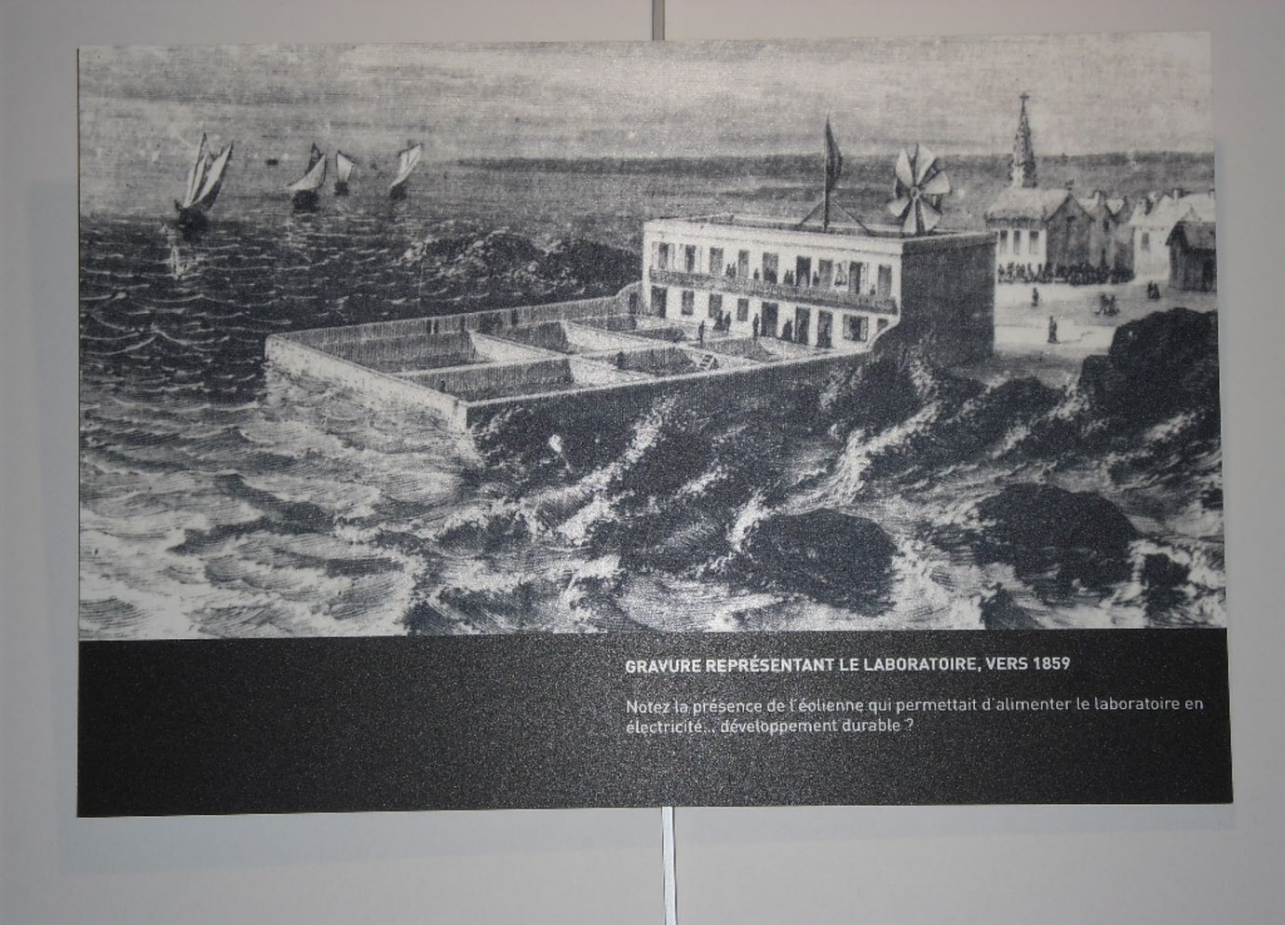
The Concarneau Marine Lab, around 1859, © Tanja Bogusz. The graph is exposed in what is now the “Marinarium” of the station. On the image is written: “See the presence of the windmill which allowed the laboratory to get electricity … sustainability?”

**Fig. 3:**
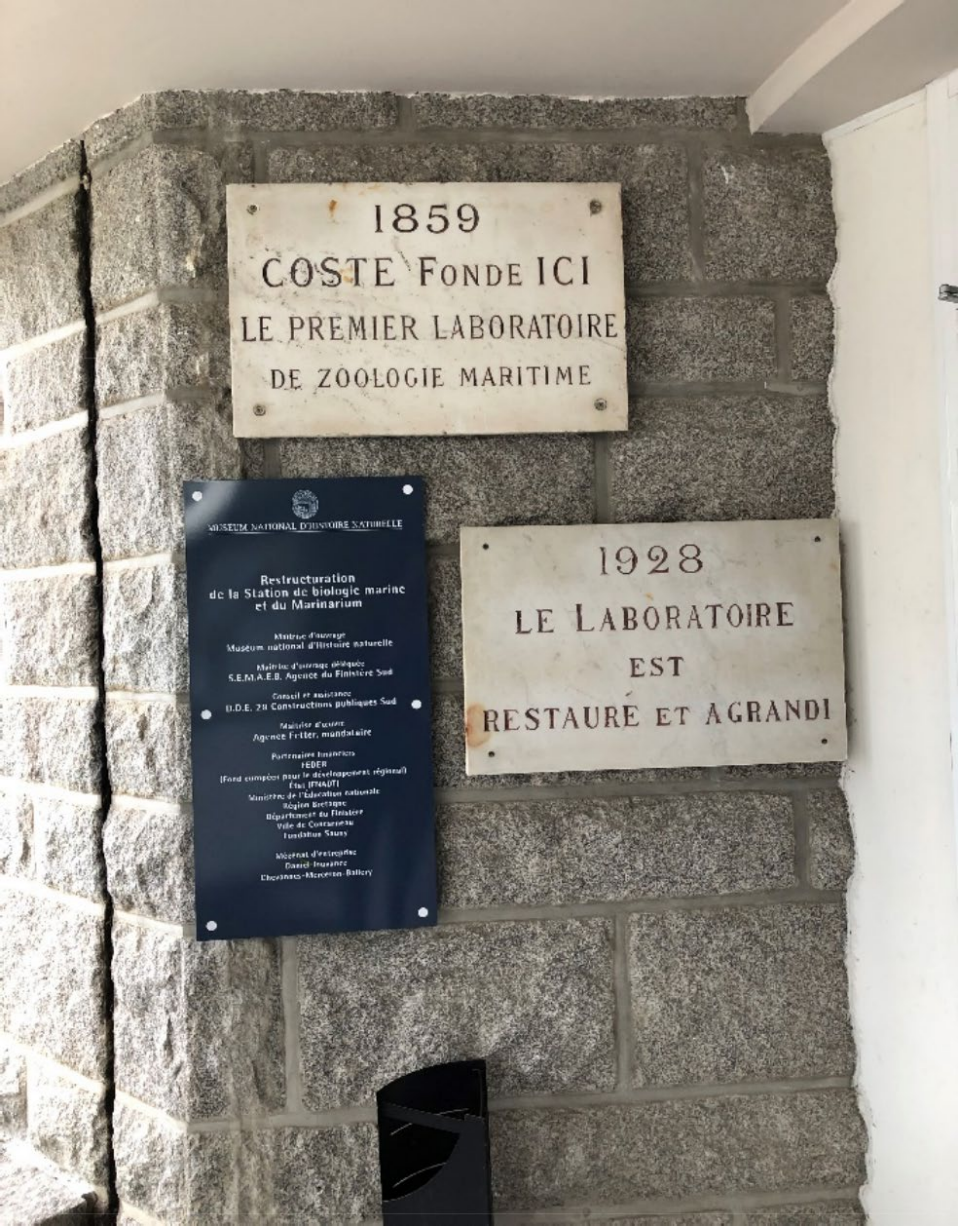
The station’s entry: A micro-history, © Tanja Bogusz

The Concarneau Marine Station in Brittany, France, is the oldest active station in the world. It was inaugurated in 1859, the same year as Charles Darwin published his “Origin of the species”–the ground-breaking oeuvre of evolution theory. This was also the year in which John Dewey, one of the key figures of American pragmatism, was born on the other side of the ocean. I will come back to him later (Figs. 2 and 3).

If the Concarneau station is the oldest active marine station worldwide, it was not the first to be established. In 1843, Belgian zoologist Pierre-Joseph Van Beneden founded the first marine station in Ostende, which was closed down at the end of the nineteenth century (De Bont, 2009, p. 202; Breine et al., 2010; Dayrat, 2016, p. 364).^3^ Sixteen years after the Ostende station’s inauguration in 1859, the Concarneau Marine Station was established by Professor Victor Coste, an embryologist who held a chair at the Collège de France in Paris. Another decade later, marine stations had grown in number, scope, and ambition worldwide. To name just those best known in the Western hemisphere, they included the Station Biologique Roscoff (France) and the Stazione Zoologica Anton Dohrn Napoli (Italy) in 1872, the Observatoire Océanologique de Banyuls-sur-Mer (France) in 1883, and the Plymouth Marine Station (UK) in 1888 (Debaz, 2005, p. 113f.). Since then, the number of stations has grown considerably worldwide throughout the twentieth century. Today, the World Association of Marine Stations (WAMS) has counted around 800, including 187 European stations^4^–each differing profoundly in terms of research focus and scope. In France, the Concarneau Station is still “often considered an inspiration for those who have succeeded” (Debaz, 2005, p. 123).^5^

**Fig. 4:**
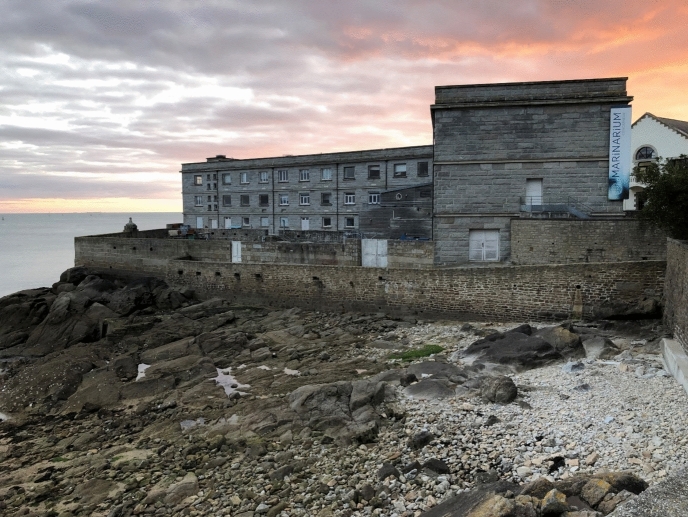
The Concarneau Marine Station today, © Tanja Bogusz

Today, the Concarneau Marine Station no longer depends on the Collège de France. It is exclusively an outpost of the Muséum national d’histoire naturelle (MNHN) in Paris. The MNHN is one of the world’s largest natural history museums and comprises a number of units within French territory. Besides Concarneau, the MNHN also hosts another station in the north of Brittany, the CRESCO Station in Dinard. Through the MNHN, the Concarneau Station is supported by the French government, the Brittany region, and the European Regional Development Fund (ERDF). Research at the station is conducted in the fields of marine biochemistry, bio-technology, ecology, and environmental management. Scientists’ surveys encompass the offshore North-East Atlantic and the polar region and are particularly sensitive to the effects of climate change on the oceans. Like many marine stations, it runs a public Marinarium (a sea-related museum), inaugurated in 1972 by the then director Yves le Gal. The Marinarium was renovated in 2023 and hosts a permanent exhibition of the local marine biodiversity, completed by thematic exhibitions on current marine-related topics. It is an important public attraction, especially for schoolchildren, and receives about 30,000 visitors every year. In line with the historically anchored museum’s engagement for nature conservation, education, and public animation (MNHN, 2022), the station organizes a number of citizen-science activities ranging from marine literacy to research-based, long-term surveys on the coastal flora, fauna, and ecology (Fig. 4).

The Concarneau Marine Station has about 50 employees, including individuals at all career levels from PhD students to professors, plus technicians and administrative staff. Two-thirds of these employees work in project-based teams. Since its beginnings, the station has been closely entangled with the IFREMER^6^–the Institut Français de Recherche pour l’Exploitation de la Mer–today known as the IFREMER Laboratoire Environnement Ressources Bretagne Occidentale (LER/BO), which has a team of eleven researchers at the station. In addition, the station hosts a division of the Brittany Institute for Agriculture Rennes-Angers, part of the French Higher Education Institution for Agriculture, Food, Horticultural, and Landscape Sciences. A special feature of the station’s outreach consists in the annual international symposium “Rendez-vous de Concarneau,” established in 2010. The symposium assembles foundational marine natural science together with biotechnology, marine engineering, and industry. The station thus engages in multiple ways with the larger region of Concarneau and Brittany through a situated mutualization (a term I will explain later) of marine knowledge.

The city of Concarneau (in Breton “Konk-Kerne”) currently comprises about 20,200 inhabitants and receives about 1,2 million visitors per year. It is a city entirely devoted to the sea–not only geographically but also economically and organizationally. Throughout its firm and vibrant maritime base, the town’s community is characterized by dense socio-professional diversity, especially within the maritime sector, thus reflecting the national situation (Bassères & Nouveau, 2022). Despite its relatively small size, Concarneau is nationally renowned for its fishing, tourism, and navy base. It hosts part of the French navy fleet and runs the European Maritime Training Centre (CEFCM), an influential navy higher education school whose graduates spread throughout the country and abroad. A large proportion of the inhabitants work in marine-related organizations and facilities. All of this makes Concarneau a truly maritime city. Marine life, knowledge, and culture are key for its inhabitants. The annual “Festival des filets bleus” (Blue Net Festival), founded in 1904, is the oldest traditional festival still existing in France. The long-standing fishery tradition experienced its heyday in the nineteenth century through the canned sardine industry; since the early 1970s it has largely turned to scallops. The *Coquilles Saint Jacques*, the object of inquiry in Michel Callon’s seminal ANT study (Callon, 1999), are also a celebrated *delicatesse* here.^7^ Following this tradition, local fisher(wo)men still represent an important economic and political power in the city.

Concarneau is also known for the “Ville close,” a fortress built in the Middle Ages and, along with the marine station’s Marinarium, today a central tourist attraction. The Ville close is surrounded by the port and the city’s fish auction hall (French: “criée”–a reference to the loud shouting while negotiating prices). Moreover, Concarneau’s advantageous situation between the Moro estuary and the Cornouaille coast is an invitation for both professional and recreational fishing, sports, and sailing, all of which enables the city to combine professional marine practices and industry with tourism. However, this is quite a lot for such a small town. During my daily conversations with residents, maritime professionals, and station members the drawbacks arose as a constant issue: They complained of rising housing prices declining local healthcare and trade services, competition for harbor space between marine leisure activities and professional fishing, and, last but not least, increasing anthropogenic pressure on the regional coast–one of the key research topics at the Concarneau Marine Station.

## Fieldwork between sea, science, and society

Despite their positionality right between the sea and coastal communities, marine stations, rather than being mere “marine laboratories” (WAMS, 2011; NAS, 2014), have been discussed within the history of science notably as “boundary objects” between the field and the lab (De Bont, 2009). However, marine stations, conducting research at cities’ coastlines, are particularly well-situated to bridge the ontological gap between “sea” and “society,” understood as two entities echoing the modern dichotomous conception of “nature” and “culture” (Latour, 1991; Descola, 2013). If the sea-society boundary has proven to be misleading given the anthropogenic impact on the ocean, current natural scientific institutions do not automatically valuate inter- and transdisciplinary forms of marine epistemologies (mundane, maritime, professional, disciplinary) on an equal footing. For example, systematic and long-term collaboration between marine natural and social scientists remains exceptional and hazardous (Bogusz et al., 2024).

However, marine stations have always contributed to instances of mutual valorization of people and the sea, and of science and society (Dayrat, 2016, p. 361; Philp, 2023). Not only have they collaborated skilfully with fishery, local stakeholders, and state institutions; from their early days on, they have also been meeting places for scientists, the public, artists, fisher(wo)men, and curious minds (Steiner, 2020). Like Concarneau, many have public aquariums, thus opening “windows to the ocean” to the public (Fischer, 2002, p. 3; Le Gal, 2009, p. 52). Climate change and biodiversity loss have also driven the governance and research of global scientific stations towards a more integrative approach: Beyond their impact on foundational research, long-term observations, data pooling, and water monitoring, field stations also “foster cross-disciplinary research communities,” as emphasized by a report from the US National Academy of Sciences (NAS, 2014, p. 2), as well as research on “scientific outposts” in general (Dumoulin-Kervran et al., 2024). Following the ongoing Decade of Sustainable Oceans, declared by the United Nations for the period 2021–2030, this potential ability of field stations makes them crucial sites for facilitating transdisciplinary research incorporating their surrounding local communities (UNESCO-IOC, 2021, p. 25).

Accordingly, given the marine imprint of the city, the Concarneau Marine Station interconnects a large variety of marine collectives. Special attention is directed towards cooperation with sustainable marine economies on the one hand, and citizen science on the other. The latter impacts on the station’s profile on various levels and is realized by means of fieldwork integrating the marine knowledge of local citizens and stakeholders. During my period of study at the station, I was impressed by the innovative way the scientists integrated artistic experimentations, as well as the local public through citizen-science activities and included these into their research methodologies (Thibaut et al., 2022). In this paper, however, I will explore other modes of collaboration–such as socio-technical and socio-epistemic–which have been examined in less detail within marine science studies. Studies on citizen science, or so-called “real world laboratories,” point to a high degree of socio-cultural homogeneity among their participants, as well as between participants and organizers (Engels & Walz, 2018; Strasser et al., 2019). In particular, projects aiming for more social cohesion, i.e., greater heterogeneity, in order to overcome social disparities through a participatory approach grapple with the problem that they seldom reach social strata that are neither academic nor driven by values represented within these strata. This misalignment seems to be linked to an implicit prerequisite that participants should share such values to be able to take part.

What does this mean for a station like Concarneau, which is, after all, locally and culturally well situated to include a multitude of marine knowledges? Before delving deeper into the two cases of socio-technical and socio-epistemic collaboration at the Marine Station Concarneau, I will present the analytical approach of my study. Taking up the pragmatist assumption on the current eco-social importance of organizing cooperation *across different social groups*, the next section explores an experimentalist approach to understanding valuation practices through what I call “heterogeneous collaboration.”

## Marine uncertainty, valuation, and the public–an experimentalist approach

Marine research today is fundamentally impacted by the planetary socio-ecological crisis caused by human activities (Druglitrø & Asdal, 2024; Folke et al., 2021). This crisis is inducing an unprecedented level of uncertainty in coastal communities worldwide. Ocean heating, plastic pollution, sea-level rise, flooding, the abundance of algae on beaches, overfishing, biodiversity loss, seabird migration, and the general disturbance of the marine ecosystem–all generate serious problems. Marine uncertainty about “matters of concern” (Latour, 2004) regularly creates target conflicts between different social groups (Neumann et al., 2017). Marine policy emphasizes, implicitly or explicitly, “environmental conscience” and the creation of a public sphere based on sustainability (Tendero, 2021). This approach is endorsed by a science-policy narrative fuelled by the hypothesis of generally shared values. In global marine governance, particularly within the framework of the UN Ocean Decade, this valuation mode is documented by an idealistic “we” rhetoric, expressed as “The science we need for the ocean we want” (UNESCO-IOC, 2021, p. 17).

Approaches based on the assumption of shared values are also echoed within the emerging field of marine social sciences (MSS). They bring together different social sciences and humanities specializing in human-sea relations (Bavinck & Verrips, 2020; Kołodziej & Kołodziej-Durnaś, 2022; McKinley et al., 2020) and explore current transitions of global marine communities, as well as modes of inter- and transdisciplinary collaboration (Bogusz, 2023; Pelke & Simonn, 2023). Although some MSS scholars are sceptical vis-à-vis valuations based on shared norms (Pauwelhussen, 2020; Peters, 2020), others rehearse the Ocean Decade’s rhetoric to emphasize, for example, “the marine social sciences we want” fuelled by support for geopolitical marine justice (Bleischwitz, 2022).

However, the shared-value-rhetoric calls for a critical, pragmatist revision. If the guiding principle underpinning the UN Ocean Decade’s “we” might work for prescriptive science-policy statements, it is, nevertheless, based on an universalistic claim that is not by any means shared by all members of marine communities. To overcome profound societal uncertainties, pragmatism has taught us that universalistic claims tend to homogenize the multiplicity of valuation framings to the detriment of what William James called “knowledge by acquaintance”–i.e., knowledge based on a variety of practice-based experiences (James, 1950, p. 221; Dewey, 1966, p. 64; Koloma Beck, 2020), opposing it to “consciousness” (James, 1912). Indeed, research has found no empirical evidence of a causal relation between environmental consciousness, practice, and impact (Hüther & et al., 2012; Diekmann & Ries, 2023; Engels et al., 2023), as implicitly or explicitly postulated by statements on science-policy governance.

To contribute to the socio-marine cohesion suggested by the “we” of the Ocean Decade, I instead propose an experimental approach to the valuation of marine knowledge, combining pragmatist experimentalism with science and technology studies (STS). Experimentalism embraces the two worlds of science and society in times of profound socio-ecological uncertainty. It seeks the creation of social cohesion without denying social heterogeneity. While STS experiments with the methodological integration of heterogenous artifacts and technology in social analysis (Law, 1987) and emphasizes the materiality of the political sphere through an “empiricist stance” (Marres & Lezaun, 2011, p. 491), US pragmatist philosophy (e.g., William James and John Dewey) has provided the empiricist epistemology. Dewey combined empiricism with evolution theory to develop what he called, in 1916, an “experimental theory of knowledge” (Dewey, 1977) from which he later deduced his “Theory of Valuation,” published in 1939. In the latter, he criticized the occurrence of both deterministic and idealistic approaches towards uncertainty, which we also observe today (Dewey, 1966, p. 1).

Dewey understood uncertainty not only as the anthropological baseline of the ever-contingent condition of life, but also as a key impulse for knowledge exploration. Uncertainty, according to Dewey, enacts experimental practice (Dewey, 2008). In this way, he established an operational link between scientific and other forms of knowledge. However, crisis situations can turn uncertainty into a problem, which is especially evident in the context of eco-social transformation. As in Dewey’s times, social transformation means unsettling acquired habits. It creates testing situations (Boltanski & Thévenot, 2006) as ways “of knowing, valuating and intervening” (Marres & Stark, 2020, p. 424) within a common good, putting social cohesion under tension. But it can simultaneously lead to unprecedented modes of cooperation. Dewey identified the preconditions for social cohesion using a conception of “democratic experimentalism” grounded in epistemology, a conception that presents cooperation as practical problem-solving intended to process uncertainty, contingency, and experiential differences in modern societies (Bogusz, 2022; Dewey, 1957).

This experimental approach transcends what has been called the “participatory turn” in science and society (Marres, 2012, p. 15) documented by the abundance of citizen science at natural history museums and marine stations. Undoubtedly, citizen science is an important tool for strengthening public attention for nature’s concerns. However, citizen science and transdisciplinarity more than often face the problem that they do not reach those who fear the consequences of protecting nature for their own livelihoods. Reaching mostly, albeit not exclusively, the “already converted,” generally well-educated cohorts, they might involuntarily even contribute to a broadening of the gulf between socially disparate groups or harden what sociologist Arlie Hochschild called the “empathy walls” (Hochschild, 2016, p. 5) between them. By contrast, an experimentalist approach based on pragmatism and STS does not precondition public participation and cooperation by means of shared values. Instead, they are facilitated by what STS scholar Susan L. Star called “cooperation without consensus” (Star, 1993). As pragmatist analyses have shown, this does not exclude the valuation of actors’ capacity to contribute to social cohesion–quite the contrary: In their neo-pragmatist social theory of normative orders, French sociologists Luc Boltanski and Laurent Thévenot have developed a corresponding analytical framework to profile the democratic implications of the absence of shared values while negotiating through conflicts (Boltanski & Thévenot, 1999, p. 374; Boltanski, 2009, p. 56), thereby updating Dewey’s concept of democratic experimentalism (Bogusz, 2014).

Valuating marine knowledge across socially heterogeneous actors is especially appealing for marine stations that are situated in the heart of dynamic marine communities comprising a large social variety of marine actors–as is the case in Concarneau. Hypothetically, these stations seem particularly suitable for contributing to heterogeneous collaborations. However, such contributions have rarely been studied in detail. As an ethnographer at the Concarneau Marine Station, I encountered two types of valuating diverse modes of marine knowledge. In order to explore such valuations, I now present these two types, based on fieldwork excursions I attended with scientists from the Concarneau Station.

## Mutualizing marine expertise: two types of heterogeneous collaboration

Marine knowledge enacted at a marine station through fieldwork is multi-faceted. Being permanently situated close to the sea implies not only a particularly physical experiential attachment to it, but also a close entanglement with the coastal community. The station’s boundary position between laboratory, sea, and society triggers particular types of encountering, integrating, and valuating activities. During my altogether five months stay at the Marine Station Concarneau between 2022 and 2024, which was dedicated to understanding the methodological approach of marine fieldwork, I was impressed by the social diversity of marine local actors and infrastructure that were mobilized to do research. Unlike expeditions or pure laboratory work, the marine station is not only a place where socio-marine interconnections are an everyday endeavour, it is also shaped by the fact that the entities, people, organizations, practices and devices involved are also “stational”–not in the sense of static, but permanent. The station’s position as an institution was tightly interwoven with its exposure–i.e., with its “feet in the water”–in the city of Concarneau, as well as in the larger region. Meanwhile, the temporal dimension played a pivotal role when I accompanied scientists during their fieldwork activities.

**Fig. 5:**
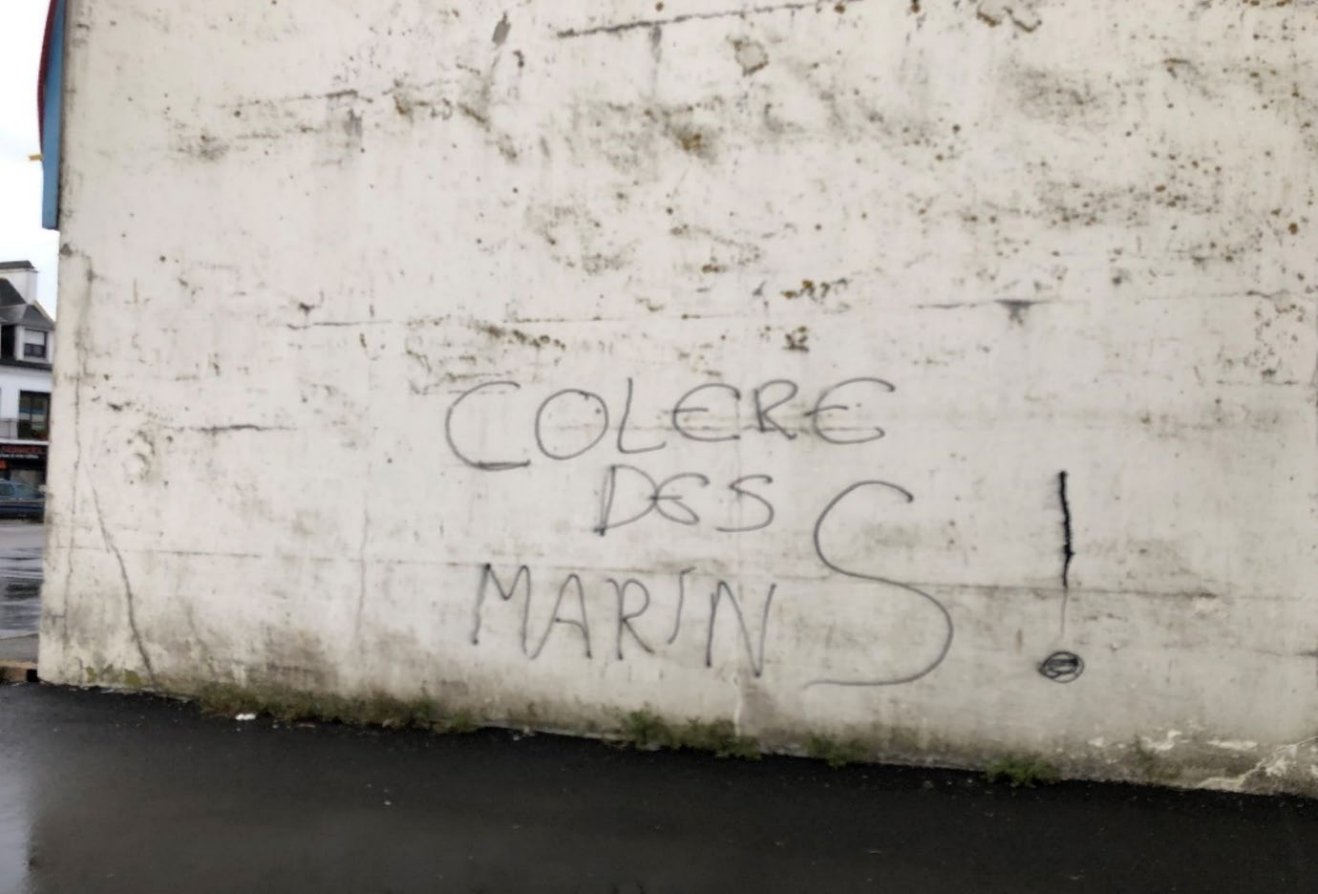
“Seamen anger!” Concarneau Auction Hall, September 2024, © Tanja Bogusz

“Being there,” in situ–terms associated with fieldwork, whether in the social or natural sciences–does not, however, mean that long and continuous presence is something that can be taken for granted, or that it happens without conflict. In an era of political polarization, natural scientists are often perceived as those who bring the bad news on the consequences of climate change and biodiversity loss, leading to legal and governmental interventions into established routines. The intense use of marine shipping routes and resources, the planning of marine protected areas, and new, sustainable industries on- and offshore make the sea not only a transmitter for social interconnections, but also a contested and embattled space. For local governments in coastal communities, it is next to impossible to equally meet the needs and interests of agri- and pisciculture, tourism, the fishery economy, leisure marine activities, and marine protection. Rather, their relative incommensurability has prompted public antagonisms within coastal communities around the globe. France is no exception. During my stay in spring 2023, the French government decided on the European implementation of marine protected areas in its coastal regions, including Brittany. Fishery organizations protested, closed roads, and constructed barricades, even setting, apparently unintentionally, the national biodiversity office in Brest on fire. I didn’t attend the protests, but their traces were still visible during my fourth and last stay in Concarneau in 2024 (Fig. 5).

However, as empirical studies on the French yellow vest movement have emphasized, “distancing from institutional environmentalism” as it is enacted through European maritime policy might be “consistent with overall distance from institutionalized politics in general and should not be considered as an opposition to environmentalism per se.” (Levain et al., 2022:, p. 557). The fisher(wo)mens’ protest against the restriction of their quota should, thus, not lead to a simplified judgement on their relation to the sea as determined through pure economic interest. Moreover, and in stark contrast to the polarizing picture of the “Britton furious fishermen,” Brittany and especially the Concarneau Finistère-region, are known for their longstanding cultivation of socially highly diverse associations (ibid, p. 562). The related term I frequently encountered was “mutualization”, which could be roughly translated by “pooling of resources”.^8^ Pragmatist scholars have related mutualization to anthropological theories of the gift and cooperation corresponding to sociological experimentalism as introduced above: First, cooperation is possible “without shared norms and values” (Adloff, 2016, p. xii); second, this activity implies “radically democratic features” (ibid.). But how could marine mutualization overrule marine polarization without a priori shared values on, for example, an ecologically sustainable ocean?

Mutualization differs from participation as enacted in citizen-science projects as follows: Both parties follow disparate interests, but they valuate the outcome of the collaboration on comparable levels regarding their respective collectives. This is commonly summed up by the proverb “one good turn deserves another.” But there is a slight difference in that the forms of sea-related mutualization I observed in Concarneau were based on non-human “boundary objects” (Star & Griesemer, 1989) acting as either the initiator or device for collaboration. In the following section, I will present such practices of mutualizing marine expertise in more detail: (a) socio-technical and (b) socio-epistemic collaboration. What these two types of collaboration have in common is that they are compiled by socially heterogeneous actors. They are thus not facilitated by shared values, e.g. environmental consciousness, but by marine knowledge interwoven with concrete entities (Levain et al., 2023): a) a vessel, and b) an octopus. As for terminology, I argue that neither “socio-technical” nor “socio-epistemic” are meant to reify classical dichotomizations–here the “social,” there the “material” etc. Rather, they respectively assume the materiality and the epistemic character, thus emphasizing their ontological entanglement, as well as their interdependence within “the social.”

### Socio-technical collaboration

Marine data collection is based on fieldwork. Historically, many marine stations were founded because of their advantageous position for exercising long-term observations of the coastal zones in which they are embedded. As marine life is ever-changing, such observations provide information about these zones’ evolution and development over decades. Given the anthropogenic imprint on marine life, today the stations have great importance for registering and possibly mitigating the degrading effects of climate change and biodiversity loss. A troubled marine ecosystem impacts coastal communities that depend on the quality of their waters. Long-term observations are thus more than foundational science. Marine scientists survey coastal transformations and give monitoring information to public authorities, industries, and stakeholders. Through their knowledge, they provide regular updates on the state of the sea and help society to understand the interactions between human activities and marine life.

At the Marine Station in Concarneau, as in several other French marine stations, such long-term littoral observations are carried out by the IFREMER. Since the 1980’s, the IFREMER has set up a priority monitoring campaign called “REPHY” (Réseau d’Observation et de Surveillance du Phytoplancton et de l’Hydrologie dans les eaux littorals), designated for the surveillance and analysis of phytoplankton in the French littoral. Marine phytoplankton is a core topic for marine knowledge, as it constitutes the baseline of the marine life cycle and produces more than 50% of our planet’s oxygen. It metabolizes the same amount of carbon dioxide as continental plants and is, therefore, an important indicator of the state of marine life facing global warming and the ecological degradation of the oceans. The REPHY campaign is divided into thematic subsections managed by different divisions within IFREMER France (IFREMER Unité Littoral, 2023). Following a selection of specific coastal sites and a fieldwork protocol, scientists are appointed to go to these sites every 14 days, take samples, and analyse them in the station’s lab. The data are entered into the French “Quadrige” database, where they can be accessed and compared by scientists and stakeholders across time and space (IFREMER Envlit, 2023). Among the MNHN stations in Brittany, two IFREMER teams are contributing to this campaign: the Concarneau Station, and the Marine Station Dinard. During my period of study, I attended four monitoring field trips from both stations, including two trips in the Bay of Douarnenez. These two Douarnenez trips, led by an IFREMER scientist based at the Concarneau Station, drew my attention to a socio-technical mode of collaboration which seemed to me particularly striking regarding the maritime imprint of Concarneau.

**Fig. 6:**
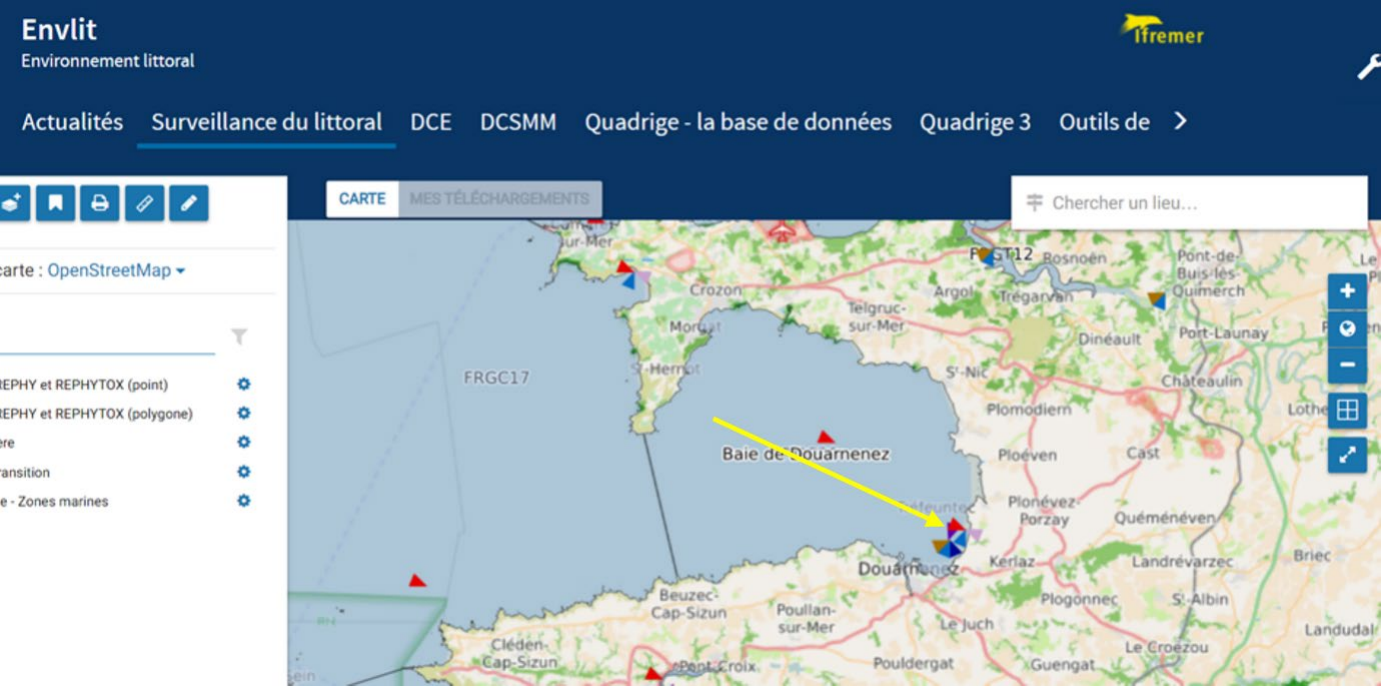
Screenshot of the coastline in Douarnenez Bay. The triangles indicate the positions surveyed by IFREMER. The yellow arrows point to the field trip described in this paper

Marine fieldwork is cost- and time-intensive–which is one reason why it has, at times, a critical standing among scientific institutions struggling for funding. Although it can–especially at a marine station–theoretically be done “on foot,” a core prerequisite of doing marine fieldwork is the availability of a research fleet, a vessel, or simply a boat. However, not every marine-station section has boats at its disposal, and this applies to Concarneau. This infrastructural need could be seen as a weakness in a station’s organizational framework, and researchers occasionally expressed their dissatisfaction about it. However, in the case of the REPHY campaign in the Bay of Douarnenez, the solution chosen reflected not only a productive way of dealing with what John Dewey defined as “the method by which warranted desires and ends-in-view are formed: by which, in short, valuation takes place” (Dewey, 1966, p. 54f), it also facilitated the encounter of marine actors who otherwise might not follow the same goals these days: (ex-)fisher(wo)men and scientists. Such encounters resulting from crisis situations are frequent in the context of socio-ecological challenges where concrete material needs of different collectives overrule their respective public antagonisms–such as the protest of coastal fisher(wo)men against marine protection based on scientific assessment. The Bay of Douarnenez thus turned out to be an intriguing starting point not only for its natural marine beauty, but also for what Noortje Marres calls “material politics” based on technical devices (Marres, 2012) (Fig. 6).

Why am I emphasizing the technological aspect here? To understand the potential scope of this kind of collaboration, it is important to note how it came into being. The story related to me was typical of what I often heard on the Britton culture of mutualization. It is not important whether it literally happened like this; it accounts for the outcome I witnessed during the field trips. The story went as follows: Several years ago, not far from the city of Concarneau, a station IFREMER member was invited to the private house of the director of the local division of the French society of coastguards, the Société Nationale de Sauvetage en Mer (SNSM). They had dinner and a few drinks. The atmosphere was cordial and relaxed. Eventually, it occurred to the SNSM director that the IFREMER lacked a research vessel to do their monitoring of the Finistère coast. And vice-versa, the IFREMER researcher realized that the SNSM lacked money to support its fleet. They became aware of their respective needs and of the complementary character of what they each had to offer. For one it was a boat and a crew to enable scientists to conduct their surveys smoothly and safely. For the other it was financial support for the navigation of his coastguards. The same night, they shook hands and a contract was born.

The SNSM is a public marine association based on the support of 9,000 volunteers, including many retired fishermen and (fewer) -women. The retired fisher crew members who facilitated the trips I attended told me that they appreciate volunteering for the SNSM because it gives them an opportunity to literally stay in touch with the sea. They felt proud to be needed: Their craft, expertise, and skill in operating and navigating the fleet are key components for marine knowledge. This valuation of experiential and professional expertise, not based on university-diploma, but on technical education, maritime skill, and often knowledge acquired over decades through seafaring was especially evident during fieldwork.

**Fig. 7:**
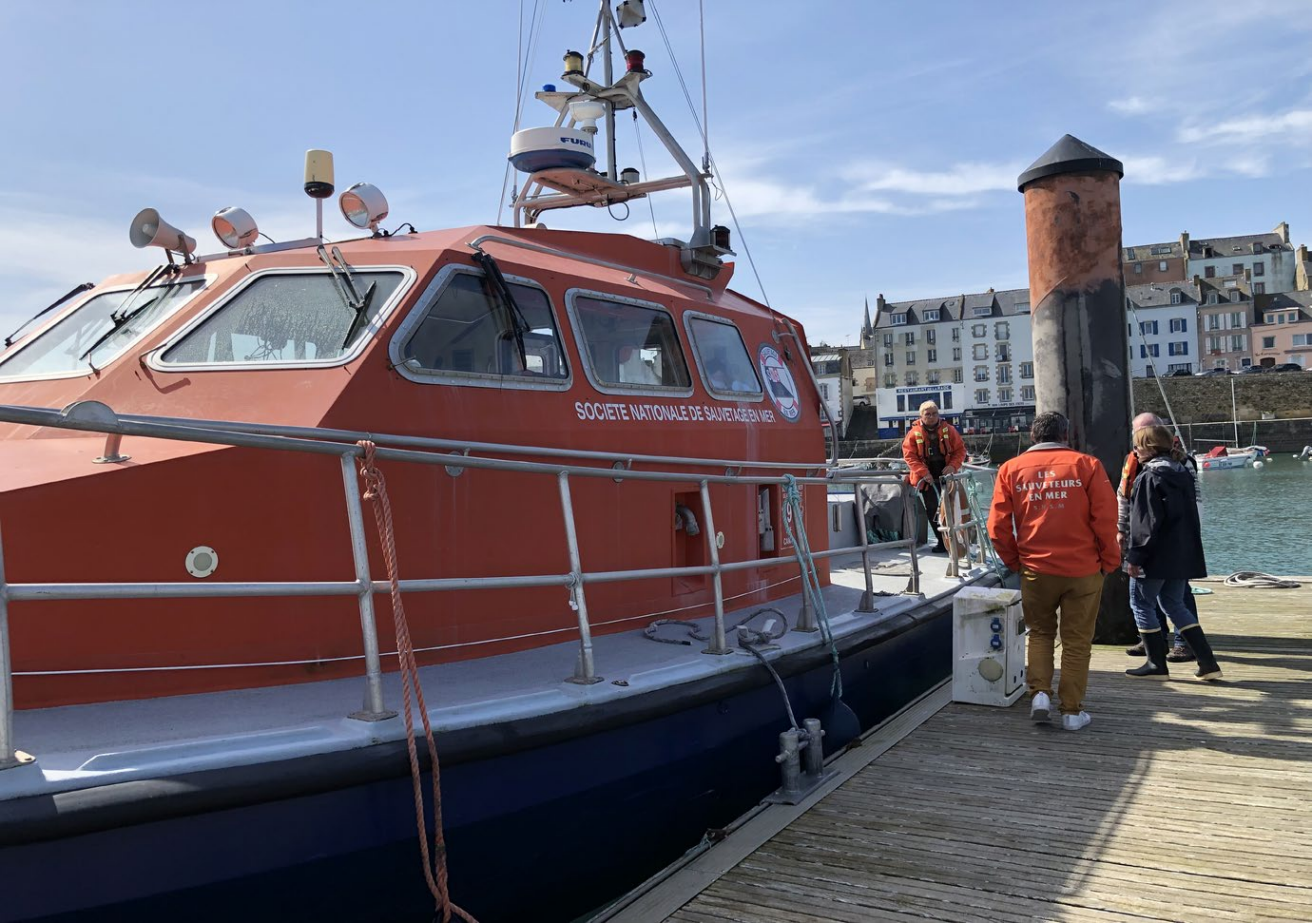
Boarding the SNSM vessel at the port of Douarnenez, © Tanja Bogusz

The sample activity follows a designated protocol which is rehearsed along the French Hexagon littoral. The researcher I accompanied went from Concarneau to the port of Douarnenez by car. When we arrived, the SNSM crew–five people probably between 40 and 70 years old–awaited us and some chat ensued: how is everyone doing, the weather, and the state of the seas. Then we boarded the vessel, which was part of the local SNSM fleet–it was a “canot de tous temps” (all-weather vessel) (Fig. 7).

**Fig. 8:**
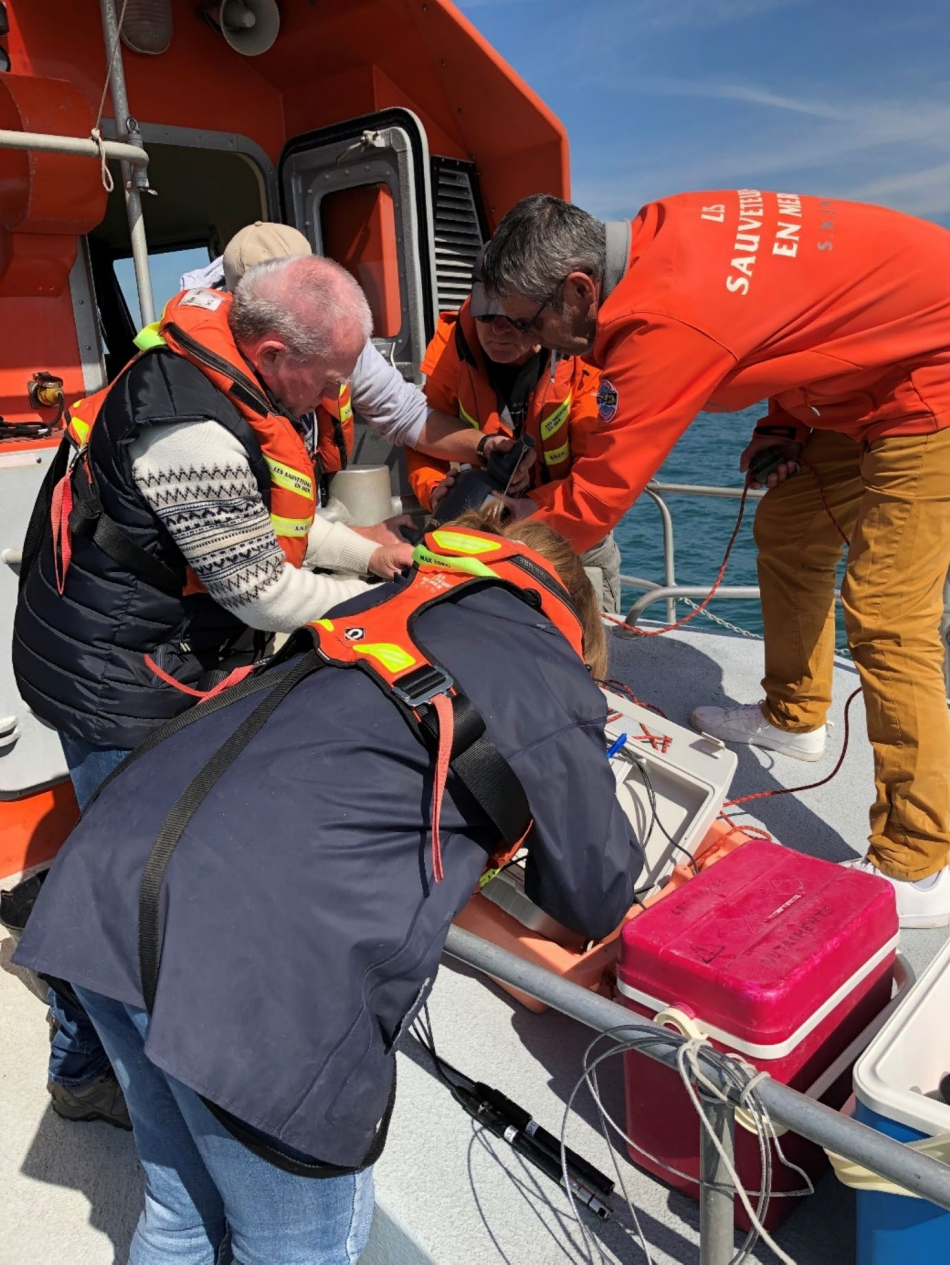
Heterogeneous collaboration between a scientist and coastguards at the Douarnenez bay, © Tanja Bogusz

The crew helped the researcher store her equipment and handed us life vests. Then the vessel set off out to sea. The samples had to be taken at a certain distance from the shore to minimize the human factor, the researcher had earlier explained to me. During the trip, a fine, long net was put out at one side of the moving vessel to collect the phytoplankton. At a certain point, the captain anchored at a specified position according to the GPS coordinates determined by the IREMER where the sample was to be taken. Then everything happened quickly: A measurement device was set up to measure the temperature, salinity, dissolved oxygen, and turbidity of the water. On the other side of the boat, the NISKIN, a 5-L bottle with a tap, was lowered into the water, then pulled up and brought onto the vessel’s deck. Following the protocol, several samples of water were taken and placed into separate small vials: some directly, others through a filter, and others again after being manipulated in situ with a substance added to the vials by the researcher immediately after extraction to keep the cells in a certain condition. All the samples were stowed in a large icebox and thus not only cooled, but also kept away from light which could otherwise affect the results. When all the samples were taken, the vessel raised the anchor and rushed back to port. The whole procedure took about 20 min. It had to be done quickly because it should always happen without drifting away from the sampling point and at about two hours from high tide (Fig. 8).

When I attended the surveys, the atmosphere on the vessel was both concentrated and cheerful. The crew keenly anticipated every single step of the procedure. I was stunned at how smoothly and quickly the workflow went, as though this heterogeneous team had been working together for decades. When a new crew member showed up, the procedural tasks involved in taking samples was explained to them by an SNSM colleague and the IFREMER scientist. They knew how to sink the sample bottle into the water and to what depth, to keep it level throughout the process of extraction and manipulation by the researcher, and to simultaneously keep an eye on the filters and measuring devices. Once the vessel was anchored at the appointed position, the captain took care not to drift away too far to keep the sampling activity on track.

The socio-technical mutualization of marine knowledge, experience, and expertise was not based on shared norms or values, but enacted via a boundary object, e.g., a vessel. It was the vessel’s capacity to assemble heterogeneous marine actors and devices, which, in turn, transcended their respective social spheres. Valuation expressed through mutualization led to a heterogeneous collaboration between science, seafaring, and sea rescue. For to collaborate successfully, a shared value related to the respective professional activities enacted by the partners was not needed (Boltanski & Thévenot, 1999). Initiated by a boundary object (a vessel fleet), however, this collaboration facilitated micro-encounters between heterogeneous actors–encounters which tend to become less frequent in times of political polarization. They provide opportunities for the mutual valuation of marine knowledge, skills, and expertise and, therefore, potential infrastructural elements for social cohesion. In a sense, as Marres and Lezaun (2011, p. 491) put it, the vessel stimulates reflection on how “objects, devices, settings and materials, not just subjects, acquire […] political capacities.” Transformed from a device to save human lives into a floating laboratory, the vessel transformed its users as well. Being acquainted with the sea for decades, the coastguards transformed their professional knowledge into marine expertise for science-related technical assistance. On the other hand, being educated as a science-technician at the IFREMER, the scientist was transformed by the vessel into an instructor for marine surveys. Yet the scientist had no control on the boat and was fully aware that no doctoral degree could overrule the coastguards’ skill in the event of danger. Eventually, the vessel itself, given its capacity to transform heterogeneous marine actors, transformed its a priori function and thereby its relation to the sea as well. No longer confined to the task of keeping onboard humans safe, it became a facilitator for a) the encounter between humans and marine organisms, and b) for the encounter of humans from different social strata.

Through the transformative capacity of the vessel, the scientist and the coastguards created a democratic moment of micro-material politics beyond the universalistic or agoristic (Marres & Lezaun, 2011, p. 497) understanding of the shared-value approach discussed above. The absence of a material device on which research at a marine station could rely led to an experimental search for support, and this created, virtually *en passant*, conjunctive, experiential ties with local marine stakeholders and community members based on mutual valorization and respect. What’s more, such socio-technical collaboration can be sustainable in that it enacts trade-offs for the partners–one vessel could fit different needs. In the following section, it is around a certain marine resident that collaboration is built. Although it resonates with Callon’s (1999) famous study in starting with the *Coquilles Saint Jacques*, at the Concarneau Station it was yet another boundary object that inspired heterogeneous collaboration.

### Socio-epistemic collaboration

Fieldwork at a marine station does not always mean going to sea. Instead, access to marine species can be provided by intermediary actors and institutions. A privileged partner for many stations and marine science in general has been fishery (Steiner, 2020), and in the Concarneau Marine Station’s history, there have been many collaborations–as well as conflicts–between fishers and scientists (Chatry, 2021). Today, however, their relation is particularly hampered by the public polarization between the maintenance of the fishery economy and the protection of marine life. During my stay in spring 2023, the protest campaign against the implementation of marine protected areas on the French north Atlantic coast, “filière morte” (“dead net”), also mobilized the fishing community in Concarneau to go on strike, to build and burn barricades in the port area, and to block the surrounding main roads.^9^

As outlined above, Concarneau is a maritime city with a long tradition of fishery. It is one of the Breton cities famed for their scallops, known colloquially as *Coquilles Saint Jacques*. For science study scholars, this name is closely linked to the seminal study by sociologist Michel Callon compiled up to the late 1970s, published in 1986, and now a classic of science and technology studies (Callon, 1999). *Coquilles Saint Jacques* are still an important economic and cultural factor in Brittany. Like Callon’s *Saint Brieuc* in the 1970s, the Concarneau scallops are confronted today by starfish predators, which, probably due to the heating of the seas, have a strong ally–or, rather, competitor–the octopus, namely *Octopus vulgaris*. However, a causal interrelation between the enormous increase in the numbers of octopus in 2021^10^ and the declining numbers of shellfish and scallops has not been confirmed. Neither has this been a favourite research topic for scientists. Even so, since 2021 the public debate on the disappearing coquilles has grown more intense. Their endangered population has created a lot of uncertainty among the fishing community. Regional and national media have documented alarming news: Fisher(wo)men have been collecting more and more empty shells and consider the proliferation of octopus, today itself an important source of income for the local fishery industry, to be the cause of the problem (France Bleu, 2021; Letailleur & Clouette, 2022).

**Fig. 9:**
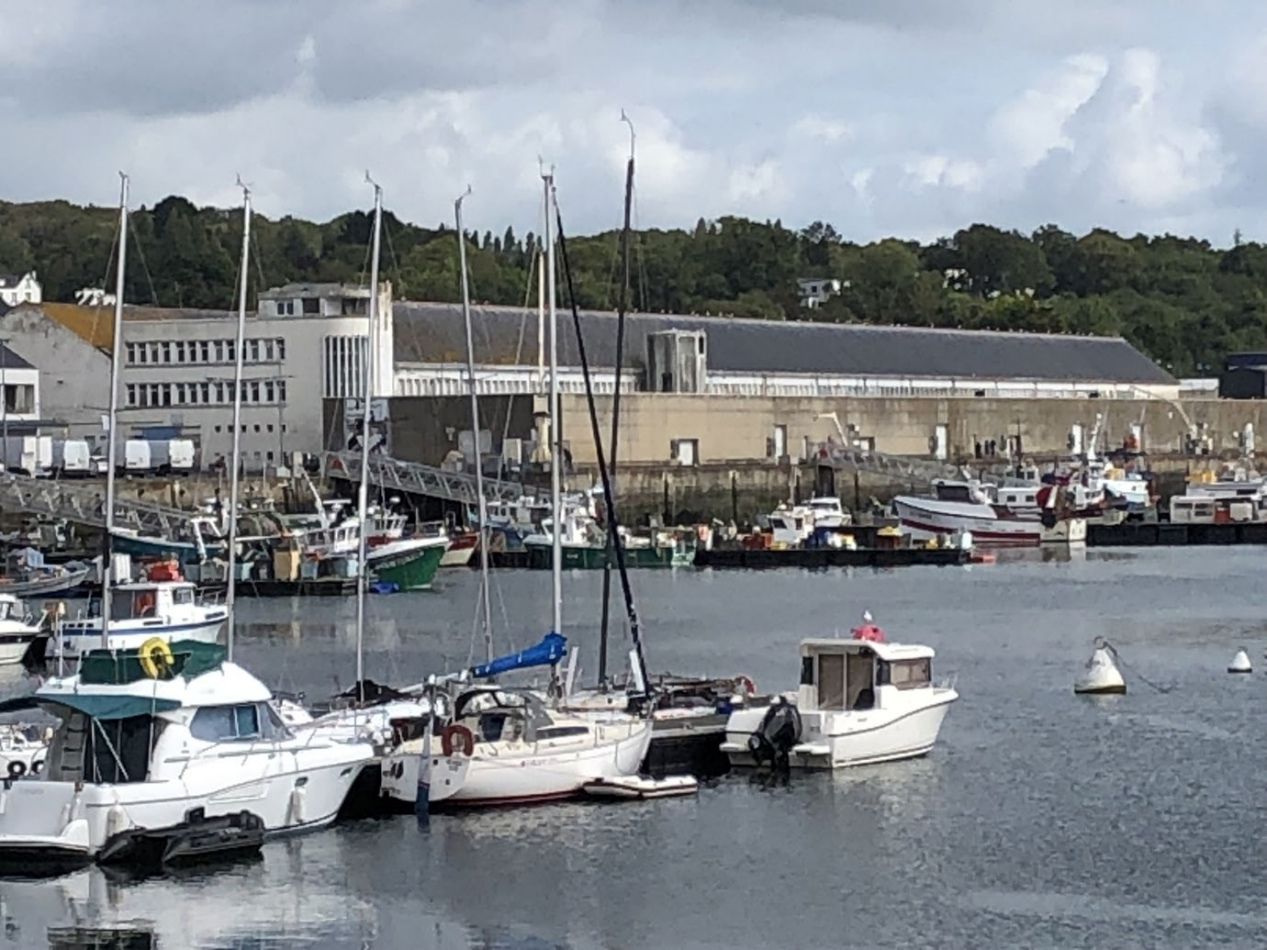
The fishery harbor and the Concarneau criée, © Tanja Bogusz

**Fig. 10:**
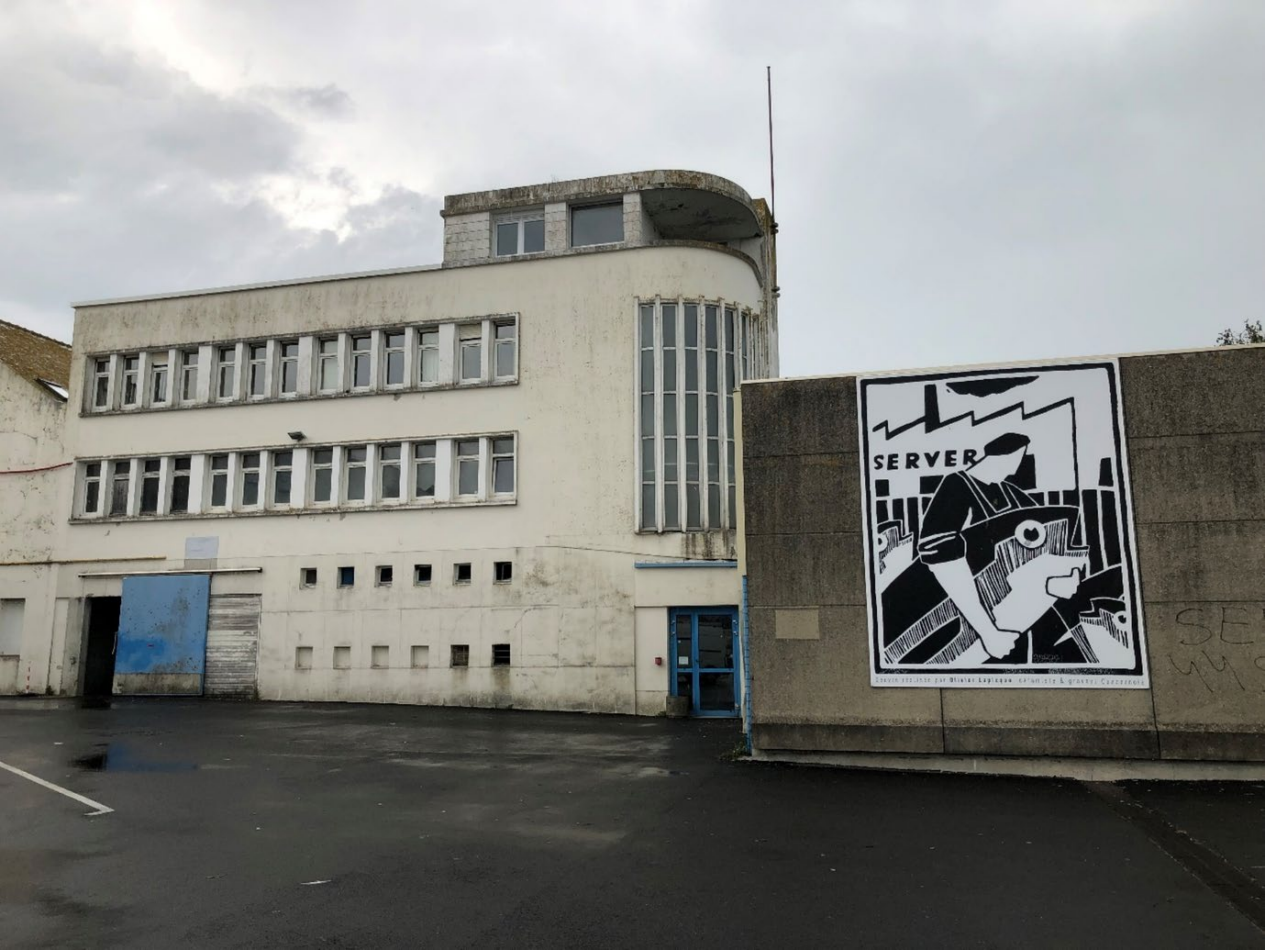
The Concarneau criée, © Tanja Bogusz

With the European funding of a research project headed by the regional fishery committee (Comité Régional des Pêches Maritimes de Bretagne) and partnered by the IFREMER and the MNHN (i.e., the Concarneau Marine Station), a group of marine biologists, along with fisher(wo)men, started to study the issue in the famously named “Poulpe Fiction” project, spanning from May 2022 until December 2024.^11^ One of the station’s project members has specialized in the biology of starfish and octopus. He invited me to attend a selection session related to the project at the Concarneau fishery auction hall, the “criée.” These assessments take place every 14 days, he explained. Yet due to the “filière morte” strike, the session was postponed. The political upheaval made me even more curious to meet the people from the fishery committee and to see how they collaborate with the station’s scientists. Eventually, the day arrived, and we went to the Concarneau criée (Figs. 9 and 10).

The hall was extremely grand–and cold. Despite the spring temperature, the station’s scientist was dressed in warm fishery clothes, including beanie and scarf. He was supported by a young intern, who helped him sort and select the octopus caught by the fisher(wo)men. In the hall, fish and other marine animals were being sorted according to their species and selling destinations and stocked in huge boxes. Staff were busy transporting them from one place to another throughout this huge facility using forklift-trucks. Two fishery representatives in yellow working clothes awaited us in a separate, also enormous, hall: The co-leader of the project, deputy general secretary of the Comité des Pêches de Bretagne and a marine biologist by training, and one permanent member of the Comité Départmental de la Pêche Maritime et des Élévages Marins du Finistère (CDPMEM 29). The *Octopus vulgaris* were placed in about 60 boxes.

How did this collaboration begin? I was told that the idea to work together had arisen several years earlier through shared acquaintances–quite similar to the above discussed IFREMER case: sitting together one night, having some drinks, discussing the topic, and translating it into a project. The abundance of octopus in Cournaille Bay, including the islands of Glénan, inclined the local fishery community to rethink their extraction practice. They not only wanted to understand whether the octopus were, along with the starfish, the source of the fall in *Coquilles Saint Jacques* stocks, but above all if and how they could adapt their fishery management to the situation. The danger of fishing the scallops too early–thus hampering their reproduction–needed a clear and general approach. Simultaneously, the management of octopus fishery was also to be adapted. The behavior and development of the octopus could therefore inform their practices, because the fishers’ navigation and extraction might also impact the octopus’ reproduction and, hence, the fate of the scallops. The fishers’ interest in applied research on the octopus was thus an *epistemic* one: it was about gaining “referred expertise” (Collins & Evans, 2002, p. 257) about the marine species in order to integrate it into their professional knowledge acquired through their fishery activity.

On the other hand, the station’s scientist was an expert not only on octopus, but also on starfish. For him, research on the octopus’ ecology could enhance foundational scientific knowledge on the interaction between the two species, as well as providing data on the interrelation between climate change, ocean heating, species migration, and species reproduction. High numbers of octopus at different states of maturation are hard to get near-shore. The ability of the fisher(wo)men to remove them from the deep sea, along with their professional knowledge based on long-term observations through their fishery-expertise, thus promised striking contextual information for understanding the species.

**Fig. 11:**
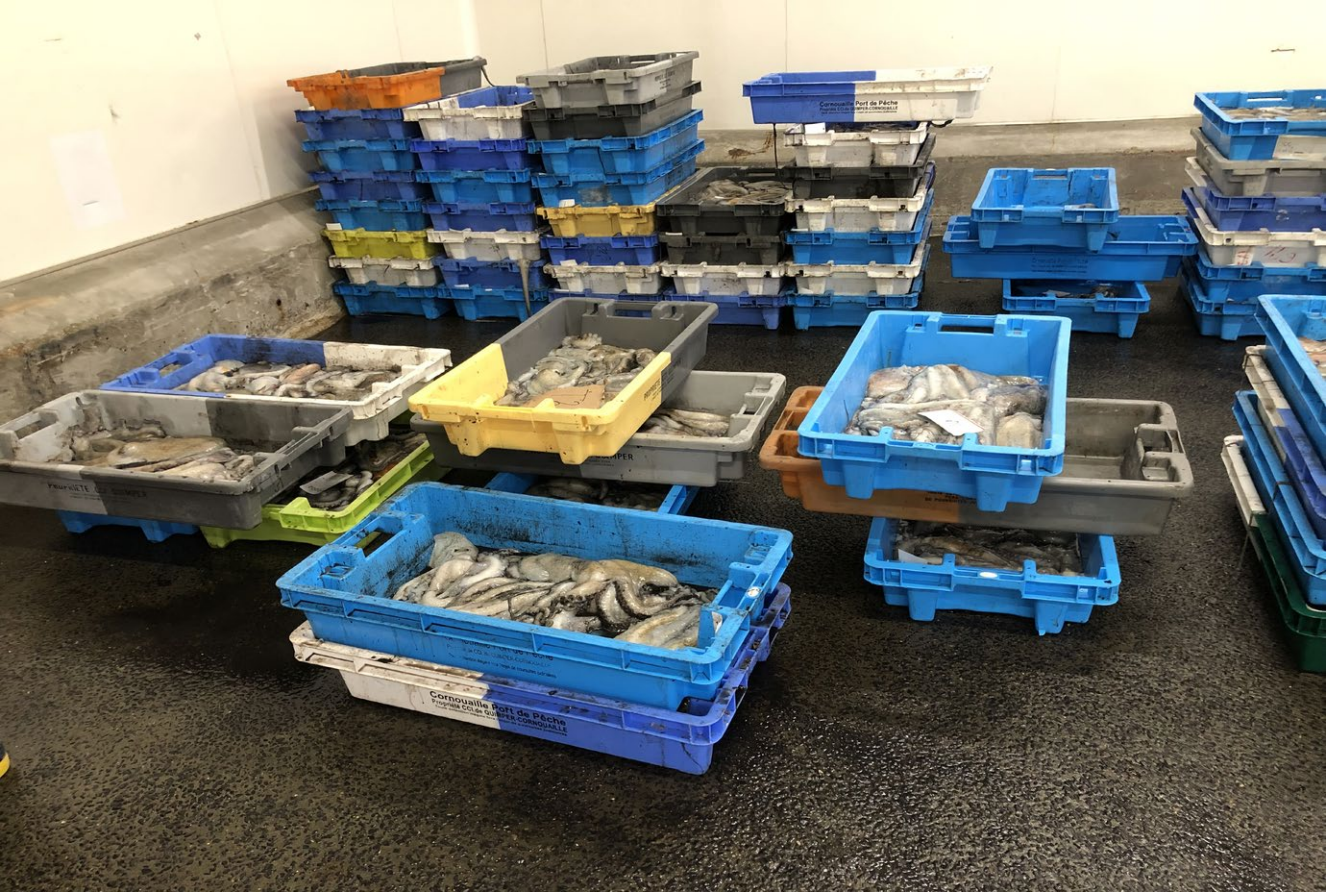
Octopus vulgaris stocked in the criée, © Tanja Bogusz

**Fig. 12:**
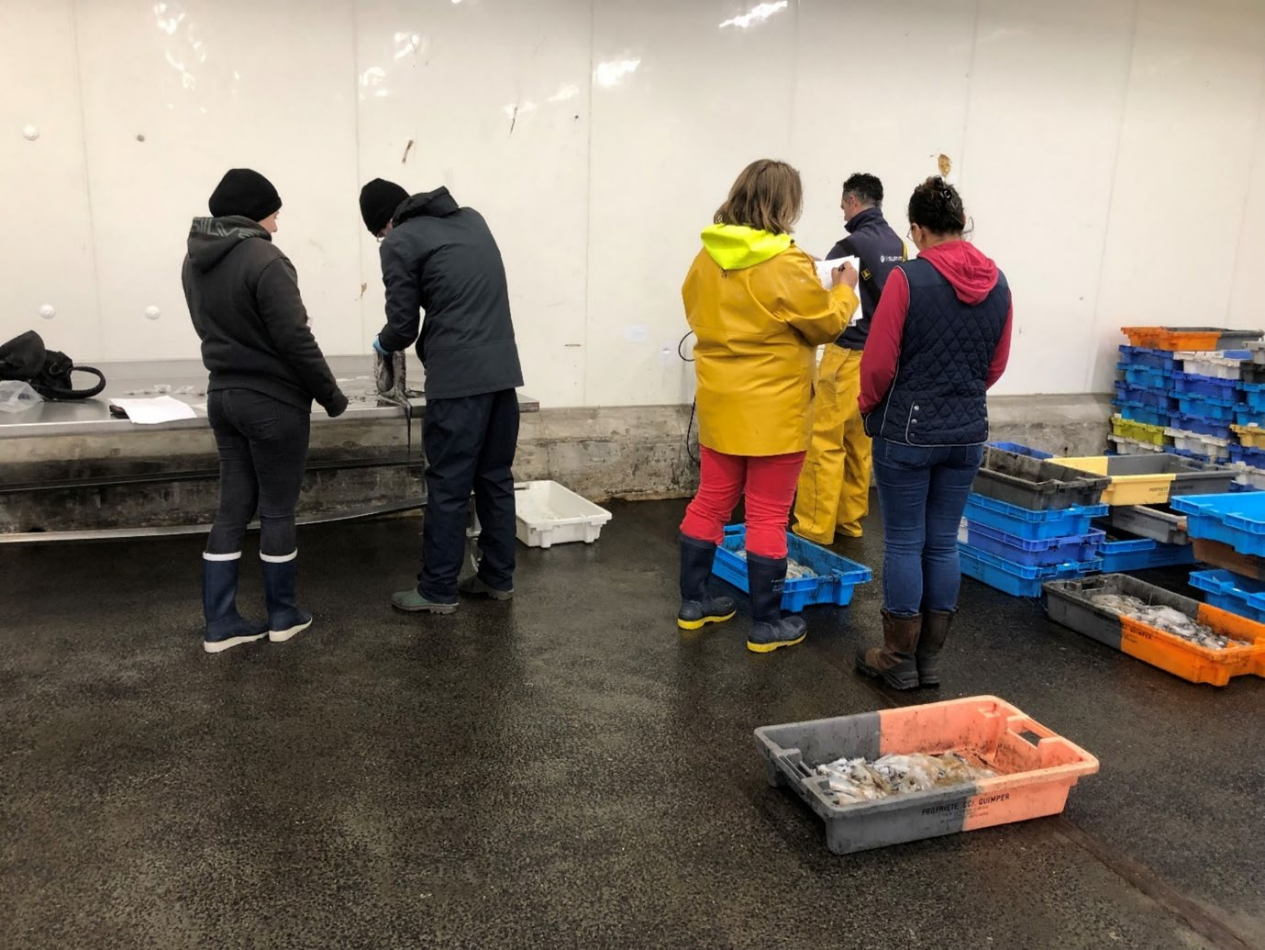
Heterogeneous collaboration between scientists and fishery professionals at the criée, © Tanja Bogusz

At the criée, the octopus were sorted by vessel and by size (Fig. 11). When the fisher(wo)men return to shore, they estimated each octopus’s weight and put them in different plastic boxes. Labels on the boxes indicated the class of weight by a number, and the name of the boat from which the species were caught. Then, two representatives from the Brittany Fisheries Committee gave us a warm welcome, and the workflow started: The colleagues from the fishery committee decided on the boxes to be examined by the station’s scientists, while the scientist chose the particular species within these boxes that were of interest for the research. Following the latter's decision, the fishery colleagues went to the balance and one of them weighed each selected octopus, measured it with a metallic ruler, and defined the sex. The other noted the information in a protocol. The octopus was handed over to the station’s scientist, who, wearing protective gloves, placed the animal on a metallic table. He sorted his extraction instruments to remove the octopus’ beak, which, he told me, could reveal the octopus’s age, in a manner comparable to the annual rings on trees. This information was added to the protocol by the intern. In the meantime, the intern prepared small plastic bags for the octopus’s beak samples with numbers corresponding to the information in the protocol. In addition, they exchanged first estimations on the determination of the species by checking a laminated sheet with photographs and visual descriptions of diverse species of octopus and the sexual maturity stages of both male and female octopuses (Fig. 12).

When the extraction and collection of the octopus’ beaks had been completed, the fishery colleagues re-stocked the remaining animals and cleaned up the hall. This involved a lot of physical work. Each action and movement showed their professional routine and skill. In between, they chatted and had some laughs with the station researcher and with each other on the findings, or on everyday topics, such as a family dinner, the weather, or things they had experienced at sea. I asked them why this work was important to them. “Normally,” they replied, “science wouldn’t give us a thought, so we are content to see that our knowledge is valuable. It’s a good thing because the research gives us a better idea of where and how to fish, so that we can adjust to our needs and use it for a sustainable fishery.”

This statement hints at the socio-epistemic transformation of the fishery-science relation enacted through the “Poulpe Fiction” project, including its co-leader who embraced both professional worlds. Moreover, the fact that the project was oriented towards applied “scientific fishery” (Steiner, 2020) echoed a longstanding tradition of heterogeneous collaboration realized within marine stations in the past (ibid.; Chatry, 2021). However, the “multidimensional knowledge transfer” (Steiner, 2020, p. 161) inspired by local acquaintance was initially not based on a shared value, as the above quote might suggest. Rather, it was incited by a marine “matter of concern” (Latour, 2004) materialized through the abundance of a non-human actor, transformed in a boundary object: the *Octopus vulgaris*. Finally, during further informal exchanges, the participants emphasized the Poulpe Fiction project’s craft of bringing together partners who are not used to collaborating. This resonates deeply with the pragmatist conceptions of cooperation discussed above (Sennett, 2012).

## Conclusion

Marine stations provide excellent field sites to study the valuation of marine knowledge in the Anthropocene for three reasons: Firstly, they are enacting “science with their feet in the water.” Secondly, they are situated in specific coastal societies, thereby enacting marine-human relations on an everyday-basis. And thirdly, they are, given their often-remote situation, connected through complex material, infrastructural, epistemic, as well as institutional layers with multiple other marine-human entities. In this paper, I have proposed a sociological analysis of valuating marine knowledge through two types of heterogeneous collaboration: technological and epistemic. Each type, explored by ethnographic inquiry and reflected through theoretical analysis (Kalthoff & Hirschauer, 2022), is based on the pragmatist assumption that socially successful cooperation can be achieved across differences, i.e., by “taking seriously a more radical diversity in how people know, value and engage with the sea” (Pauwelhussen, 2020, p. 2). Crucially, both the above-described socio-technical collaboration, and the socio-epistemic collaboration, emerged via local marine entanglements. In both cases, the parties involved were an integral part of the local marine community. At the same time, they were not associated through shared values. According to the experimentalist approach outlined above, the absence of shared values is not an impeding factor for heterogeneous collaboration. Quite the opposite: As the two presented cases indicate, heterogeneous collaboration can be built and consolidated around a materiality, a technology, an infrastructure, or even a marine species.

Adding to the two cases discussed, a third type of heterogeneous collaboration can be envisioned for further examination, this time across the natural marine sciences and the social and cultural marine sciences and humanities. In contrast to often time-limited, project-based approaches, such interdisciplinary collaboration advocates for long-term marine knowledge exchange consolidated by permanence. Marine stations could act as incubators for such collaboration. The study of current mutualization practices (Van Assche et al., 2020, p. 3) and the possible consequences of future sea-society challenges for social cohesion (Sweet, 1998) call for an interdisciplinary approach in the spirit of “contributory expertise” (Collins & Evans, 2002), which could be provided by the social sciences. Furthermore, interdisciplinary collaboration needs a firm footing on methodological, organizational, and educational levels. Integrating the social and natural marine sciences, and the implementation of interdisciplinary marine research as part of academic training, are key for a successful realization of interdisciplinary collaboration (Bogusz et al., 2024).

This paper assumes that, despite the structural increase in social disparities, affecting even small and generally economically well-off communities such as the city of Concarneau, heterogeneous collaboration could include actors from different socio-economical strata. It is thus potentially also efficient in terms of social cohesion, as these types of valuating marine knowledge incite mutualization. The observation of such collaborations invites for giving more “attention to everyday life and routine work as important locations where the practices of politics, science, and technology meet.” (Druglitrø & Asdal, 2024, p. 3). Uncovering these mundane micro-politics through ethnographic studies can reveal “practices that de facto preserve the environment without it being necessarily politicized.” (Levain et al., 2022, p. 566). They reveal the methodological limits of the shared value approach for four reasons: *First*, cooperation based on social difference increases social cohesion (Durkheim, 1999). *Second*, the shared value approach, as exemplified in the statements of the Ocean Decade, rehearses a universalistic approach on the political sphere, where (human) actors representing three ontological entities–sea, science, and society–are expected to agree, while the foundational eco-systemic crisis of the ocean urgently calls for an empiricist and pragmatic reflection. *Third*, the shared-value-approach crucially underestimates the socio-structural imprint of values such as “sustainability” and risks overcharging and, therefore, excluding those who do not share them from the outset (Crosman et al., 2022). *Fourth* and finally, the universalistic stance does not consistently account for the mutualizing impact of non-human actors in the valorization of marine knowledge, which might enable heterogenous actors to collaborate eye-to-eye.

The UN Ocean Decade Implementation Plan has formulated ambitious goals to face current and future sea-society challenges: “Throughout the Ocean Decade, natural and social scientists and ocean stakeholders will work together to co-design and co-deliver solution-oriented research that spans all aspects of the ocean, including human interactions, ocean–atmosphere interactions and the land-sea interface” (UNESCO-IOC, 2021, p. 17). Regarding the related targeted outcome (No. 7), the plan calls for an “inspiring and engaging ocean where society understands and values the ocean in relation to human well-being and sustainable development” (ibid., p. 19). However, relying on citizen science and universalistic shared values alone will not keep pace with the foundational eco-social polarization of the current transformation challenges. Marine stations today can play a pivotal role here: Due to their emplacement and permanence, they are experienced in enacting heterogeneous collaborations on an everyday basis. Yet, although there some 800 marine stations are currently active around the world, too little attention has been paid to such a cross-sectoral, interrelated opportunity for valuating marine knowledge in the analysis of marine stations.

The current era is profoundly characterized by increasing social disparities and uncertainties. Instead of relying on a presupposed “we” to develop socio-marine cohesion based on shared values, this paper has explored practices of mutualization between different actors through an empiricist account. Such mutualization, as observed in the maritime city of Concarneau, can serve as an example for the valorization of diverse marine knowledge, experience, and expertise. Current marine policy and research tend to undervalue such collaboration based on socio-economic heterogeneity. “However,” say Nathalie Steins and colleagues regarding the fishery sector (2020, p. 137), “collaboration between fishers and scientists typically takes place at lower (national) levels, tends to highlight specificities and detailed local knowledge and creates appreciation for heterogeneity in the local context.” Through ethnographic observation, the focus can thus be directed towards these often-invisible cross-sectoral practices of mutualizing marine knowledge that could, potentially, reduce both disparities and polarization. Locally embedded and often invisible, they might, in turn, enhance social cohesion across interdependent entities. Much needed in the current era, such enhancement echoes pragmatists’ assumptions on what John Dewey called “democratic experimentalism” (Dewey, 1957). Consistently, David Stark emphasizes in his study on valuation in economic organizations that a “society recognizes its potential when it truly gives recognition to a multiplicity of ways of defining what is valuable.” (Stark, 2011, p. xviii).

However, the limits of the research presented here are also evident: This case study concerns just one marine station by means of an “empirical paradigm” (Elias & Scotson, 1994) inspired by the pragmatist philosophy of science. However, it can instil applied research as well. Through multi-sited marine-station studies of different scales, further empirical inquiry could test and, possibly, consolidate the outlined theoretical analysis and description, as well as the methodological approach. By including quantitative methods, systematic comparative investigations of marine stations could assess these historically and epistemically rich and highly differentiated facilities in order to explore confined yet generalizable types of heterogeneous collaborations. This might, in the long run, contribute to socio-marine cohesion in a range of coastal communities. The Concarneau Marine Station, being the oldest of its kind, could thus serve as a model to profile these communities’ capacities to valuate and anchor multiple entities and actors connected to marine knowledge.

## Footnotes

1 See the film here Stations marines: la science les pieds dans l’eau | CNRS Le journal (posted by the CNRS 18.04.2023; accessed 25 July 2023).

2 Or, rather, a “seascape” (Brown & Humberstone, 2015).

3 The exact circumstances and the date of the closing of Ostende seem unclear. Breine et al. (2010) say that the Ostende Station “was in use from 1843 till 1879,” whereas Dayrat contends that the Ostende Station was “abandoned in 1895” (Dayrat, 2016 , p. 264).

4 See Endnote 2.

5 All quotes from French are my translation, with the support of Theodore Best.

6 Victor Coste, first director of the Concarneau Station, was also nominated imperial general inspector for maritime coastal fishery, the nucleus of the latter IFREMER, by Napoléon III in 1862 (Chatry 2021, p. 17ff.).

7 The MNHN-CRESCO-Marine Station in Dinard is only 80 kms from Saint Brieuc, the location of Callon’s scallop-study (Callon, 1999 [1986]).

8 During informal discussions on mutualization in Brittany, people mentioned the large number of registered associations (80,558 associations in 2021) as an indicator. Indeed, within France, Britton stands for an outstanding vibrant association-culture (Communiqué de Presse Région Académique Bretagne, 2021).

9 During my stay, which was dedicated to marine biological fieldwork methodologies, I was unfortunately not aware of the importance of the fisheries’ protest for the present account of my ethnographic inquiry. I therefore didn’t observe the protests, nor interviewed people involved in it.

10 Some scientists also consider a correlation between the decrease of octopus fishery during the pandemic and the increase of their population since 2021: La pêche miraculeuse du poulpe, un filon en or pour les professionnels de la mer, mais jusqu'à quand? (bfmtv.com); accessed 29 August 2023.

11 For a general overview on the project: https://www.cdpmem56.fr/poulpefiction/, accessed 2 January 2025.
